# Cook County

**Published:** 1874-11

**Authors:** 


### Hospitals.

***Cook County Hospital.*** Surgical Clinic. Dr. FREER. Aug. 4, 1874.

*Carcinoma—Orchitis—Phymosis—Amputation of left index finger.*

The amputation of the finger was done because of necrosis, the patient having had his fingers severely squeezed. Ether was given, and Esmarch's bandage applied above the elbow. Two lateral flaps were made, the outer one being the larger flap; the incisions being made an inch above the head of the metacarpal bone, through the dorsum of the hand. To preserve the symmetry of the hand, the head of the metacarpal bone was cut off with the forceps, obliquely from without inwards.

The patient with phymosis had a mixed chancre, a bubo in the right groin, and syphilitic roseola. The under surface of the penis was excoriated, swollen and red. Passing a director beneath the upper surface of the prepuce, it was divided quite down to the corona. Dr. Freer remarked, with much drollery, that this was another case of so-called water-closet disease; the patient said he knew of no other cause for his disease.

*Impermeable Stricture—Retention of Urine, relieved by the Aspirator—Perineal Section.*

B. L., a man of sixty-four years of age, and of great obesity; had contracted gonorrhœa twenty years ago, from which a stricture resulted. He had no trouble from this till four years ago, when he began to think he had the gravel and cystitis. Before Dr. Freer saw him, an unsuccessful attempt to pass a catheter had caused two false passages in the urethra. The urine came away guttatim; there was retention for seventy-two hours. Fomentations and cathartics were given, and a mixture containing eight drops of laudanum with fifteen drops of tinct. belladonna, every four hours. The bladder was emptied twice by the aspirator, the needle being inserted in the median line, an inch above the pubis. To allay the vesical irritability, there being quite a sharp cystitis, a drachm of spts. mindereris and thirty drops of tinct. hyoscyamus were given every three hours; also injections of bismuth, per rectum. The operation of perineal section was done under ether, the patient being in position as if for lithotomy. The stricture was divided, a catheter tied in, and directions given to have the bladder washed out with lotions of carbolic acid.

August 11. Dr. Freer.

*Amputation of the right thigh—Esmarch's Bloodless Method—Amputation at the left shoulder joint.*

H. G., forty-two years of age, Irish, while attending to his duties as a switchman, was run over by a train of cars, and suffered a compound comminuted fracture of both bones of the left leg, near the knee, and punctured wound of the lower part of the thigh, the wheels passing obliquely from right to left over the thigh, and stripping off the lower two inches of the periosteum from the femur. The operation was done twenty-one hours after the accident; the patient being under ether thirty-six minutes. Dr. Freer remarked, that for years railroad surgery had become distinct from that of most other injuries, on account of the gre and of the general destruction of the vitality of the parts, where, apparently, little harm had been done. In this hospital no limbs had been saved, when amputation had been performed below the point of contact with that which caused the injury. In applying the rubber-band, after Esmarch's method, the limb was first wrapped in oiled silk, to prevent the soiling of the bandage. The amputation was by lateral flaps, and in the middle third of the thigh. While dressing the stump, the attention of the class was called to the superior usefulness of whisky to that of the carbolic acid wash as a lotion for dressing of the stump.

The amputation at the left shoulder joint was rendered necessary by the great laceration of the entire extremity by a railroad accident. The deltoid muscle was, fortunately, well preserved, and made an excellent flap. The muscles on the inner aspect of the limb were contused higher up than those of the outer aspect The head of the bone was first turned out of the glenoid cavity, and a posterior flap made; then entering the soft parts, the operator followed up the course of the axillary artery, and ligated it; the second flap, containing the deltoid muscle, was next made.

Ununited fracture of the left clavicle, of nine weeks' duration, in which the ends overlapped, was treated by the use of Brainard's drill. The boring was done in two places, both pieces of bone being pierced. Fox's apparatus was applied, and was made out of a piece of new muslin, two yards long and one-half yard wide, —a very simple but perfectly effective and inexpensive treatment.

*Hæmorrhoids.*

This patient tells us that these hæmorrhoids have given him great inconvenience for the past six years. They are now as large as a walnut, are mostly internal, and distinct from the integument. Five large tumors were removed, the patient being under ether, by means of the clamp and the actual cautery, heated to a white heat. The sphincter ani was first dilated, so that the parts to be removed could readily be drawn down and out.

During the month, Dr. Steele has had two labor cases in which the women gave birth to twins. In the first case it was the fourth labor, and lasted seventeen hours. The presentation was vertex in the first position. The children were born two hours apart; there was one placenta for the two females. In the second woman, the labor was normal; one placenta; presentation was vertex and footling; two females; and the second labor.

